# Imaging of cell-based therapy using ^89^Zr-oxine *ex vivo* cell labeling for positron emission tomography

**DOI:** 10.7150/ntno.51391

**Published:** 2021-01-01

**Authors:** Yutaka Kurebayashi, Peter L. Choyke, Noriko Sato

**Affiliations:** Molecular Imaging Branch, Center for Cancer Research, National Cancer Institute, National Institutes of Health, Bethesda, MD, USA

**Keywords:** Zirconium-89, Zirconium-89 oxine, cell tracking, positron emission tomography, cell-based therapy

## Abstract

With the rapid development of anti-cancer cell-based therapies, such as adoptive T cell therapies using tumor-infiltrating T cells, T cell receptor transduced T cells, and chimeric antigen receptor T cells, there has been a growing interest in imaging technologies to non-invasively track transferred cells *in vivo*. Cell tracking using *ex vivo* cell labeling with positron emitting radioisotopes for positron emission tomography (PET) imaging has potential advantages over single-photon emitting radioisotopes. These advantages include intrinsically higher resolution, higher sensitivity, and higher signal-to-background ratios. Here, we review the current status of recently developed Zirconium-89 (^89^Zr)-oxine *ex vivo* cell labeling with PET imaging focusing on its applications and future perspectives. Labeling of cells with ^89^Zr-oxine is completed in a series of relatively simple steps, and its low radioactivity doses required for imaging does not interfere with the proliferation or function of the labeled immune cells. Preclinical studies have revealed that ^89^Zr-oxine PET allows high-resolution *in vivo* tracking of labeled cells for 1-2 weeks after cell transfer both in mice and non-human primates. These results provide a strong rationale for the clinical translation of ^89^Zr-oxine PET-based imaging of cell-based therapy.

## Introduction

The last decades of development in cell-based anti-cancer therapies has been marked by breakthroughs such as adoptive transfer of tumor-infiltrating T cells (TIL) 1, 2, tumor antigen specific T cell receptor (TCR)-transduced T cells (TCR-T) 3, 4, and chimeric antigen receptor T (CAR-T) cells 5-9. Adoptive transfer of TIL in melanoma patients achieves objective responses of 50 - 70 % and complete responses of 22 % when a lymphodepleting preparative regimen is employed before transfer 1, 2, 10, 11. CD19-directed CAR-T cells achieve durable complete responses in 40 % of refractory diffuse large B cell lymphoma and in 70 % of follicular lymphoma. However, some severe, sometimes fatal, side effects, including cytokine release syndrome that correlates with tumor burden and encephalopathy, have been observed 9, 12. Along with the evolution of CAR-T cells with improved intracellular signal transduction to enable proliferation and cytokine production of T cells 9, CAR technology has been expanded to other cell types such as B cells and NK cells 13. Other major breakthroughs have been made in the field of stem cell therapies. In addition to traditional hematopoietic stem cell transplants, mesenchymal stem cells or cells differentiated from induced pluripotent stem cell (iPSC) developed from the patient's own cells or embryonic stem cells have been actively investigated for the therapy or regeneration of damaged tissue 14, 15.

Common to all the cell-based therapies is their dependency on the delivery of transferred cells to the desired target in order for the cells to exert therapeutic effects. Even in the case of CAR-T cells that have enhanced target antigen recognition capabilities, the cells first have to migrate to and then infiltrate the target (*e.g.* cancer) tissue in order for them to recognize the specific antigen and exert cytotoxicity 16-18. The accumulation of transferred tumor-targeting lymphocytes can vary among the metastatic lesions within the same patient as a result of different evolutionary and/or immunoediting processes unique to each metastatic lesion 19, 20. In most clinical cell-based therapies, biopsies or observations of clinical outcome have been the method to evaluate the migration of transferred cells to the target tissue. Even if tumors are biopsied, transferred lymphocytes often cannot be distinguished from endogenous tumor-infiltrating lymphocytes unless transferred cells are labeled in advance 21, 22. The number of cells reaching the target are often quite small. Therefore, imaging of transferred cells is clinically important in order to non-invasively and quantitatively evaluate the homing of the cells to the targeted cancers but also to use as a tool in designing next generation cell therapies with better homing characteristics.

Technically, transferred cells can be tracked using single-photon emission computed tomography (SPECT) or positron emission tomography (PET) when the cells are labeled with appropriate radioisotopes. Indium-111 (^111^In)-oxine or Technetium-99m (^99m^Tc)-hexamethylpropylene amine oxime (HMPAO) scintigraphy/SPECT has been a classical method to visualize leukocytes and is clinically used for evaluation of inflammation, infection or abscess since the mid-1970s 23-26. ^111^In-oxine scintigraphy/SPECT has been previously used to analyze the distribution of adoptively transferred TILs in patients 21, 22. However, the sensitivity of SPECT is 2 to 3 orders of magnitude lower than that of PET 27, and relatively high doses of radioactivity are required for imaging, which cause death or dysfunction in the labeled cells 28-30. ^99m^Tc has a short half-life (6 hours) and is not suitable for tracing the transferred cells for multiple days, and cell labeling with ^99m^Tc-HMPAO is relatively unstable 30, 31. Alternatives to radiolabeling include magnetic resonance imaging (MRI) using superparamagnetic iron oxide nanoparticles (SPION) that are phagocytized by the cells to be tracked 32. While MRI provides detailed anatomical information without ionizing radiation, it is cumbersome to survey the entire body and also difficult to detect the signal loss caused by iron oxide unless pre- and post-SPION loading studies are compared. This poses practical concerns as well. Moreover, quantitation of SPION's dark signal void is difficult. One of the recent developments in the tracking of cells with MRI is to use Fluorine-19 (^19^F) labeling. Here, the MRI unit is tuned to the resonance frequency of ^19^F, which blocks out all the background signal from water protons. However, this method requires specialized ^19^F detection coils, and because of limitations in sensitivity, large amounts of ^19^F have to be introduced to the cells or tissues to generate detectable signals 32, 33.

In general, PET is superior in image quality, sensitivity, spatial resolution, and quantification to SPECT 27, which makes it an attractive modality for tracking cells. Among positron (β)-emitting radioisotopes used for PET, ^89^Zr has a relatively long half-life (3.3 days), ideal for tracking cells for several days, and relatively low positron energy that is required for high resolution in PET 34, 35. Therefore, PET imaging using ^89^Zr has been gaining attention, and the usefulness of ^89^Zr-immunoPET, in which antibodies or antibody fragments are labeled with ^89^Zr (*e.g.* ^89^Zr-labeled anti-PD-1/PD-L1 antibody), for imaging human patients has been evaluated 36-39. For the specific task of tracking adoptively transferred cells for multiple days with relatively high resolution, cells to be tracked are labeled with ^89^Zr-oxine and imaged by PET 40, 41. Here, we briefly review the current status of cell labeling and the applications of ^89^Zr-oxine PET imaging technique for cell-based therapy.

## Methods to track cells *in vivo* by PET imaging

Radionuclides that have been used for *ex vivo* cell labeling to longitudinally evaluate the distribution of the cells of interest *in vivo* include; Fluorine-18 (t_1/2_ = 109.7 minutes), in the form of ^18^F-fluorodeoxyglucose (FDG) to label cells *ex vivo* before transfer 42, Copper-64 (^64^Cu, t_1/2_ = 12.7 hours), such as copper-64-pyruvaldehyde-bis (N4-methylthiosemicarbazone) (^64^Cu-PTSM) 43, and ^89^Zr and Iodine-124 (t_1/2_ = 4.2 days), in the form of radio-labeled antibodies against specific cell surface proteins 44. ^18^F-FDG depends on glucose uptake by the cell and thus is not suitable for dormant cells 45. ^18^F-FDG also suffers from relatively rapid efflux after labeling, due to dephosphorylation of ^18^F-FDG-6-phosphate, the phosphorylated form of ^18^F-FDG that will be trapped within the cell 42, 45, 46. ^64^Cu-SPION has been recently developed as a PET-MRI multi-modal imaging nanoparticle that has potential benefits for tracking cells *in vivo*, taking advantage of both the high sensitivity of PET and detailed anatomical information of MRI 47. However, the half-life of ^64^Cu, although longer than that of ^18^F, is still insufficient for tracking cells beyond several days after transfer. On the other hand, ^89^Zr, with a half-life of 3.3 days, is ideal for cell tracking for 1-2 weeks. Its relatively lower positron energy improves PET spatial resolution and thus, is most often used for studies of this type.

In *in vivo* cell labeling, a radioactive labeling reagent (e.g. radioisotope-conjugated antibodies, antibody fragments, or engineered antibodies for immunoPET) is systemically administered and the distribution of the target molecule expressed on the endogenous cell populations in the body is evaluated 36-39. Thus, *in vivo* labeling cannot discriminate adoptively transferred cells from the endogenous cells expressing the same target molecules (*e.g.* adoptively transferred CD8 T cell versus endogenous CD8 T cells). Therefore, although *in vivo* cell labeling is useful for analyzing the distribution of endogenous cell populations 38, 39, it is not suitable for the evaluation of the fraction of adoptively transferred cells that reach the target tissue. *In vivo* labeling is frequently associated with high background signal due to specific or non-specific antibody distribution (*e.g.* liver and spleen) and clearance (*e.g.* renal clearance of antibody fragments), long circulation time in the blood and pooling in tissue fluid, including those caused by enhanced permeability and retention (EPR) effect in some tumor tissues 48. *In vivo* labeling also requires highly specific antibodies with little to no cross-binding to non-target cells; however, specific cell markers, although present on many T and B cells, are frequently unavailable for many other cell types such as dendritic cells or monocytes.

If adoptively transferred cells are transduced with a PET reporter gene, it is possible to administer a radioactive reporter probe to locate the transferred cells in the body 49. Since the expression level of the reporter gene is maintained after cell proliferation, reporter gene imaging can achieve longer-term tracking of transferred cells. It also detects only live cells. On the other hand, the requirement for an injection of the radioactive probe and clearance of unbound probe at each interested time point limits short-term or frequent monitoring of cell migration. Thus, monitoring cellular responses to additional stimuli that cause relatively rapid changes of cell distribution could be difficult. The biggest disadvantage of PET reporter gene imaging is its requirement for gene introduction to the cells. It is imperative that such reporter genes are carefully chosen to avoid immunogenicity. Although PET reporter gene imaging is feasible for therapeutic cells that undergo genetic engineering during their production, such as CAR-T cells, for cells that do not require genetic engineering, which are the majority of TILs and stem cells, reporter gene imaging strategies would be difficult to translate into the clinic.

In contrast to *in vivo* labeling, cells can be labeled with radioisotopes *ex vivo* before adoptive transfer to patients 40, 41. Since only the cells adoptively transferred are labeled with radioisotope, *ex vivo* labeling has essentially zero background signal in the recipient, thereby achieving extremely high signal-to-background ratios even with low cell labeling doses, and, therefore, high sensitivity to even tiny clusters of labeled cells 40, 41. Monitoring relatively small cell distribution changes to additional stimuli could be possible with this method. *Ex vivo* labeling can accurately track adoptively transferred cells. However, some background signals may develop over time due to phagocytosis of damaged labeled cells or release of the radioactive label after death of the labeled cells (*e.g.* accumulation of ^89^Zr within bone as described below). As each cell division results in approximately halving of the label per cell, image intensity can decrease due to cell division alone 40.

## Labeling of cells with ^89^Zr-oxine for PET imaging

Cell labeling by ^89^Zr-oxine is completed in three relatively simple steps that can be performed in aqueous solution 40: 1) synthesis of ^89^Zr-oxine by mixing ^89^Zr-chloride with oxine followed by neutralization at room temperature. ^89^Zr-chloride can be generated from ^89^Zr-oxalate produced by a cyclotron 50, 2) incubation of cells with synthesized ^89^Zr-oxine for 15 minutes at room temperature or at 4 ^o^C, and 3) washing of the labeled cells. The ^89^Zr-oxine synthesis yield is as high as >98 %, which, together with the aqueous conditions, eliminates the need for a purification step. With application of the generated ^89^Zr-oxine solution to a cell suspension, virtually all the cells are labeled with ^89^Zr-oxine. The synthesis and labeling steps are similar to those of ^111^In-oxine complex and thus, ^89^Zr-oxine cell labeling could be easily translated to the clinic. Recently, a slightly modified method of generation of ^89^Zr-oxine, which uses ^89^Zr-oxalate instead of ^89^Zr-chloride, has been reported 51. Another reported method of synthesis uses neutralized ^89^Zr-oxalate to react with oxine in chloroform, followed by recovery of the synthesized ^89^Zr-oxine from chloroform phase by evaporation and re-dissolving the obtained ^89^Zr-oxine in dimethyl sulfoxide for further dilution in aqueous buffer for cell labeling 40, 41.

Mechanistically, the lipid solubility of ^89^Zr-oxine allows it to diffuse through the cell membrane. Therefore, labeling with ^89^Zr-oxine does not require active uptake by cells and can be performed in resting cells and at 4 ^o^C 40. This characteristic also eliminates the requirement for expression of certain cell surface molecules for labeling or an antibody specific cell surface protein.

Experience with ^111^In-oxine is instructive. 8-hydroxyquinoline (oxine) can be released from ^111^In-oxine after the molecule enters the cytoplasm, and the free ^111^In forms a stable complex with cytoplasmic and nuclear proteins 52. The formation of ^111^In-protein complex is presumably achieved through exchange of ^111^In from 8-hydroxyquinoline to intracellular proteins 52. Free 8-hydroxyquinoline is then exported from the cell. Although a similar mechanism can be assumed for ^89^Zr-oxine, the exact mechanism of cell labeling using ^89^Zr-oxine is still unclear. Our preliminary results have shown that ^89^Zr-oxine also labels the cell membranes (cytoplasmic and intracellular membranes) as well as cytoplasmic, and nuclear proteins, while negligible binding was observed with chromatin and cytoskeleton proteins (unpublished data). Although ^89^Zr-oxine can label a variety of cells, the labeling efficiency (percentage of ^89^Zr incorporated to the cells per added activity) differs among cell types 40. This may be due to the difference in the amount of intra-cellular proteins available for ^89^Zr binding. ^89^Zr-oxine labeled cells, especially non-dividing cells (*e.g.* matured dendritic cells), retain ^89^Zr very well over multiple days 40.

In contrast to the cell-permeable ^89^Zr-oxine, ^89^Zr-deferoxamine-NCS has been reported to directly conjugate ^89^Zr to cell surface proteins for cell tracking by PET 53, 54. In this method, deferoxamine chelates ^89^Zr, while the NCS group covalently binds primary amine groups of membrane proteins on the cell surface. Therefore, ^89^Zr radioactivity is mainly detected in the membrane fraction of labeled cells but not in cytoplasmic and nuclear fractions 53.

## Maintenance of immune cell function after labeling with ^89^Zr-oxine

Importantly, labeling of immune cells including lymphocytes, macrophages, and bone marrow-derived dendritic cells with ^89^Zr-oxine does not affect their function when optimal labeling doses were used. For example, labeling with^ 89^Zr-oxine does not interfere with the activation, proliferation, and cytokine expression (IFN-γ and IL-2) after TCR stimulation in labeled CD8 T cells 40. Similarly, labeling of CAR-T cells by ^89^Zr-oxine does not affect their viability, cytotoxicity, or cytokine production 51. Labeling with ^89^Zr-oxine does not interfere with LPS-induced activation of dendritic cells and their ability to present antigens and activate T cells 40. The only exception observed to date, is that ^89^Zr-oxine labeling of bone marrow cells, even at low radioactivity doses (10.9 kBq/10^6^ cells), showed initial delay in the proliferation after granulocyte colony stimulation factor (GM-CSF) stimulation *in vitro* 55. However, the differentiation capacity of these labeled bone marrow cells is maintained both *in vitro* and *in vivo* 55. Based on these studies, the optimal labeling doses minimize cellular toxicity while being sufficient to image by PET are approximately 11 - 44 kBq/10^6^ cells, depending on type and condition of the cells.

Once labeled with ^89^Zr-oxine, ^89^Zr is stably retained in labeled cells 40. However, death of labeled cells results in rapid release of free ^89^Zr. *In vivo*, free ^89^Zr is excreted from the kidney; however, a small fraction of free ^89^Zr may be taken up in bone matrix hydroxyapatite 40, 55, 56. This small uptake in bone can be reduced by administration of deferoxamine, a chelating agent clinically used for treatment of iron overload and hemochromatosis 55. Once bound to deferoxamine, the ^89^Zr is rapidly excreted in the urinary tract.

## Monitoring of transferred cells using ^89^Zr-oxine PET in mouse models

**Figure 1:**
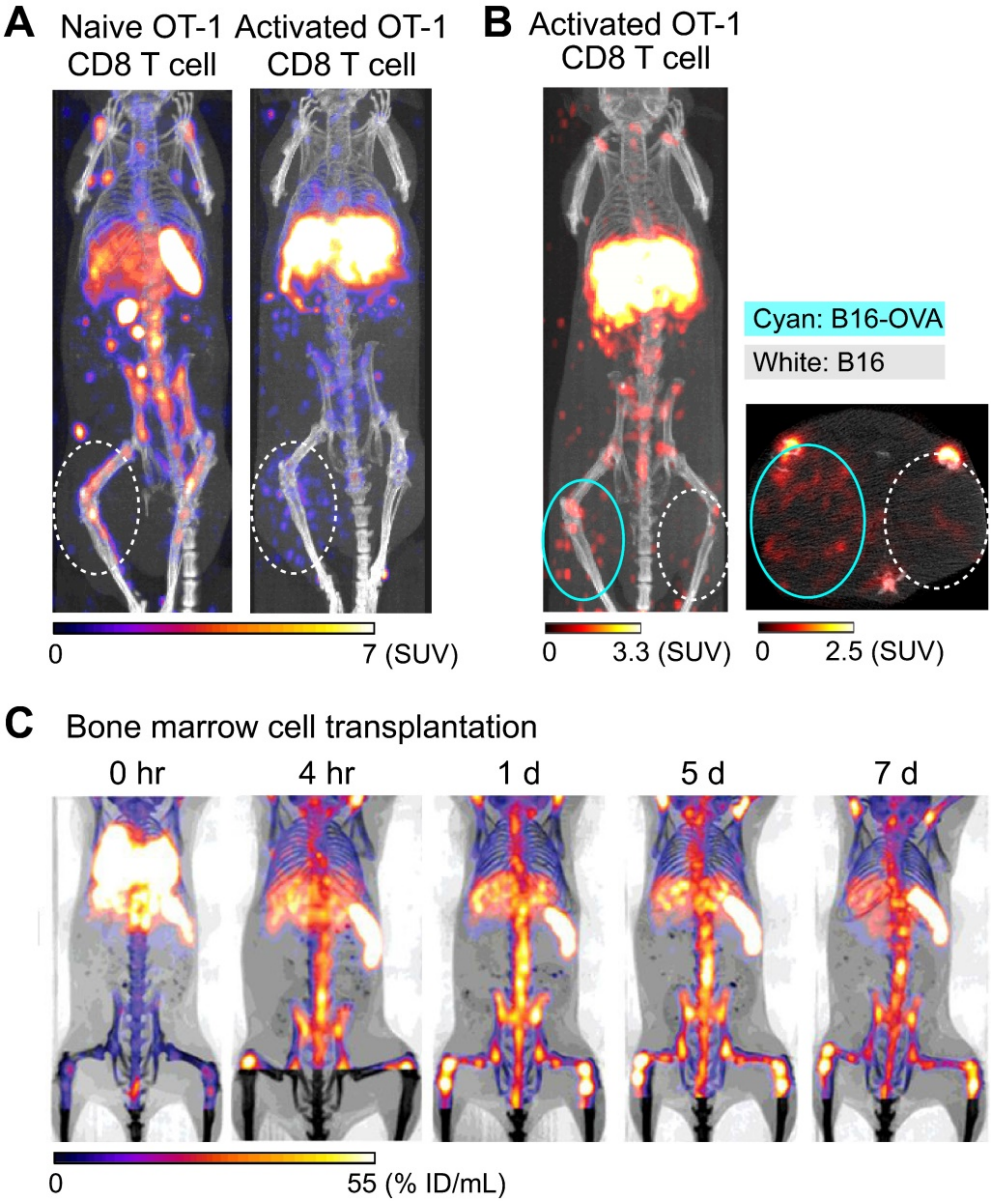
** Tracking of transferred cells using ^89^Zr-oxine PET in murine models.** A) Activated OT-1 CD8 T cells distribute to antigen-expressing B16-OVA tumor (white dashed circle), while naïve OT-1 CD8 T cells do not. Naïve OT-1 CD8 T cells (left) and activated OT-1 CD8 T cells (right, adapted from 40) were labeled with ^89^Zr-oxine (222 kBq/8 x 10^6^ cells and 248.5 kBq/7.7 x 10^6^ cells, respectively) and intravenously transferred to tumor-bearing recipient mice. PET/CT images acquired on 2nd day after transfer are shown. Also note the different distribution between naïve and activated OT-1 CD8 T cells outside the tumor. B) Activated OT-1 CD8 T cells distribute to antigen-expressing B16-OVA tumor (cyan circle) but not to antigen-negative B16 tumor (white dashed circle). Activated OT-1 CD8 T cells labeled with ^89^Zr-oxine (185 kBq/8 x 10^6^ cells) were intravenously transferred to the recipient. MicroPET/CT images acquired on 4th day after transfer are shown. Left: maximum intensity projection MIP PET/CT, right: transverse plane. C) Distribution of transferred bone marrow cells. Donor bone marrow cells were labeled with ^89^Zr-oxine (16.6 kBq/2.0 x 10^7^ cells) and intravenously transferred to the recipient. PET/CT imaging was performed at indicated time points after transfer, with multiple intramuscular injections of deferoxamine to prevent accumulation of free ^89^Zr within bone. Transferred bone marrow cells initially distribute to the lungs, followed by distribution within 4 hours to the bone marrow, liver, and spleen. Adapted from 55.

Preclinical studies have shown that ^89^Zr-oxine PET can track adoptively transferred cells over a period of 1-2 weeks. For example, adoptive transfer of ^89^Zr-oxine-labeled activated OT-I CD8 T cells (TCR transgenic T cells that recognize ovalbumin: OVA) shows initial rapid accumulation in the lungs, followed by gradual accumulation within antigen-expressing tumor (B16-OVA) by Day 2 (Figure 1A, right) 40. In contrast, these activated OT-I CD8 T cells do not accumulate in antigen-negative B16 tumors. Uptake is restricted to B16-OVA tumors, which is associated with a rapid anti-tumor effect (Figure 1B). In addition to the antigen-expressing B16-OVA tumor, ^89^Zr-oxine PET shows that activated OT-I CD8 T cells also distribute to the liver after adoptive transfer, compatible with previous observations that activated CD8 T cells preferentially home to the liver 57, 58. On the other hand, ^89^Zr-oxine PET demonstrates that naïve CD8 T cells preferentially distribute to the spleen and lymph nodes instead of the liver (Figure 1A, left). Therefore, ^89^Zr-oxine PET clearly captures the different behaviors between naïve and activated CD8 T cells after adoptive transfer. Similar results have been observed when ^89^Zr-oxine-labeled CAR-T cells are used, wherein transferred prostate stem cell antigen (PSCA)-targeting CAR-T cells accumulate in the liver as well as the PSCA-expressing tumor, after initial distribution to the lungs 51.

Vγ2Vδ9 subtype of γδ T cells, which represent most circulating γδ T cells in humans, can recognize phosphoantigens expressed on tumor cells and exert anti-tumor activity 59. When *ex vivo* expanded human Vγ2Vδ9 T cells are labeled with ^89^Zr-oxine and transferred to tumor-bearing SCID mice, ^89^Zr-oxine PET shows accumulation in the tumor and liver, after transient distribution to the lungs. 60. This can be detected by ^89^Zr-oxine PET and is augmented by administration of aminobisphosphonate, which increases the expression of phosphoantigens on the surface of tumors 59, 61.

Tracking of bone marrow cells also reveals interesting patterns. ^89^Zr-oxine PET shows the initial transient distribution of transferred bone marrow cells to the lungs, rapidly followed by homing to the bone marrow, spleen, and liver within 4 hours of transfer (Figure 1C) 55. Since the homing of bone marrow cells is dependent on CXCL12-CXCR4 axis 62, ^89^Zr-oxine PET shows delayed homing of labeled donor bone marrow cells to recipient bone marrow after administration of plerixafor, a CXCR4 antagonist 55. Co-administration of plerixafor and G-CSF further disturbed this homing process 55. On the other hand, ^89^Zr-oxine PET shows that the initial homing of transferred bone marrow cells is not affected by prior whole body irradiation 55. In a bone fracture model, ^89^Zr-oxine PET further shows the mobilization of pre-transferred ^89^Zr-labeled bone marrow cells from pre-distributed organs (bone marrow, spleen, and liver) to a site of fracture within 1 day of injury 63. This re-distribution of pre-distributed bone marrow cells is also impaired by plerixafor treatment 63. These reports indicate that ^89^Zr-oxine labeling of highly radiosensitive bone marrow could be performed with minimum effect on functionality of the cells.

With the ^89^Zr-deferoxamine-NCS direct membrane protein conjugation method, human mesenchymal stem cells have been imaged, showing their distribution to the lungs and liver after intravenous injection in athymic nude mice 53. Interestingly most human mesenchymal stem cells labeled with ^89^Zr-deferoxamine-NCS remain within the injected site for at least one week after injection into myocardium in athymic nude mice 53.

## Monitoring of transferred cells using ^89^Zr-oxine PET in non-human primates

**Figure 2:**
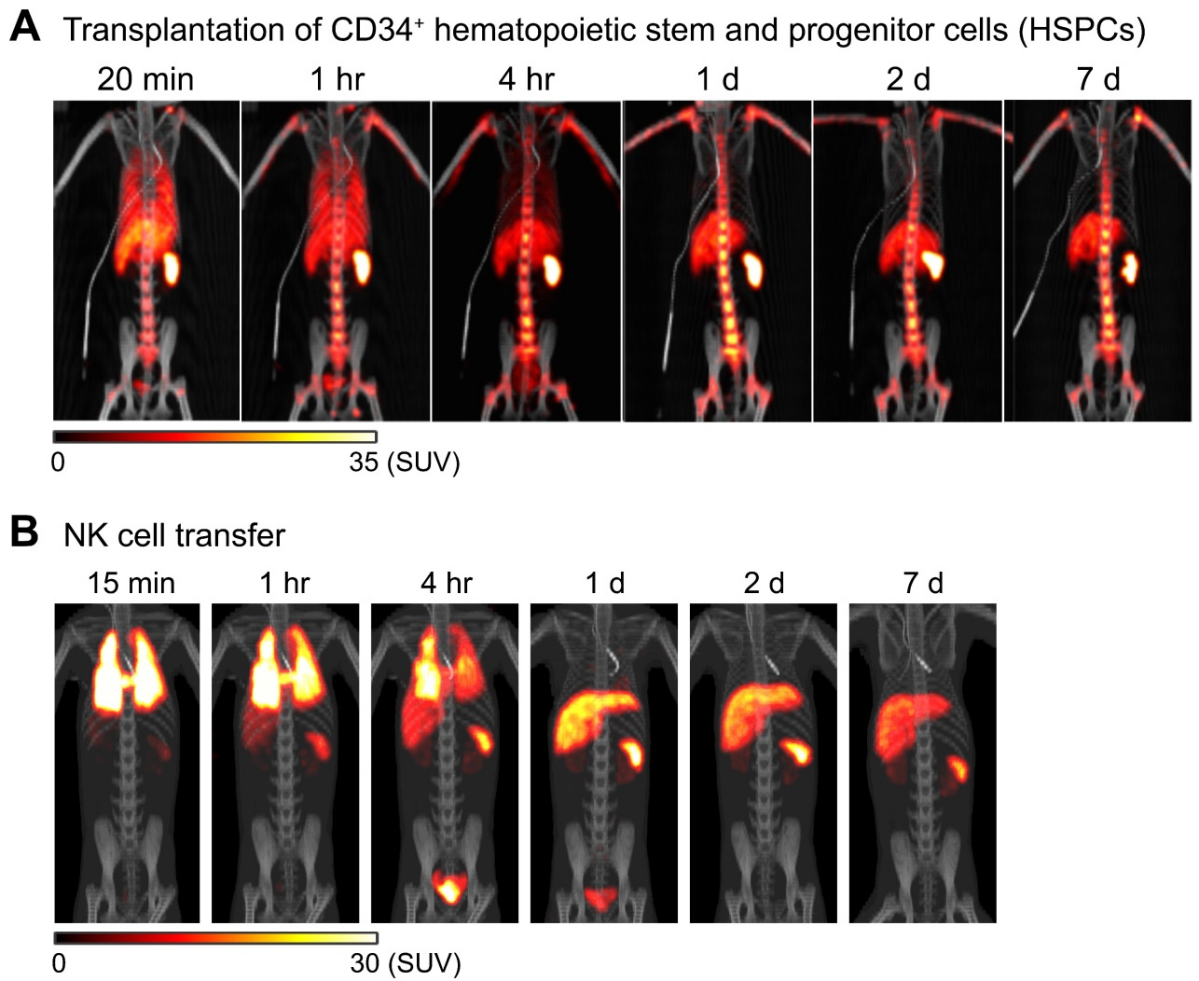
** Tracking of transferred cells using ^89^Zr-oxine PET in non-human primate models.** A) Distribution of transferred CD34^+^ hematopoietic stem and progenitor cells (HSPCs) in a rhesus macaque. CD34^+^ HSPCs mobilized by plerixafor and G-CSF treatment were labeled with ^89^Zr-oxine and autologously transferred intravenously (44.4 kBq/10^6^ cells, 1.2 x 10^6^ cells/kg). PET/CT was performed longitudinally under continuous infusion of deferoxamine to prevent accumulation of free ^89^Zr within bone. Transferred cells rapidly distribute to the bone marrow, liver, and spleen 64. B) Distribution of transferred NK cells in a rhesus macaque. NK cells purified from peripheral blood were expanded *ex vivo* with IL-2, labeled with ^89^Zr-oxine, and autologously transferred intravenously (15.2 kBq/10^6^ cells, 21.9 x 10^6^ cells/kg). Cell migration was longitudinally tracked by PET/CT. Deferoxamine was continuously infused during the entire imaging study. Transferred NK cells initially distribute to the lungs, followed by gradual distribution to the liver and spleen. Little distribution of NK cells to the bone marrow is observed. Adapted from 64.

^89^Zr-oxine PET can be used to image adoptively transferred cells in non-human primates. For example, in an autologous intravenous hematopoietic stem cell transplantation model in rhesus macaques, ^89^Zr-oxine PET imaging shows that CD34^+^ hematopoietic stem and progenitor cells (HSPCs) accumulate in the bone marrow, liver, and spleen after rapid passage through the lungs (Figure 2A) 64, similar to the pattern seen in a murine model of bone marrow transplantation (Figure 1C) 55. Clinically, intrabone transplantation of HSPCs, especially cord blood cells, has been suggested as a method to improve engraftment in the recipient's bone marrow 65, 66, and preclinical studies using swine and macaque were undertaken to determine if homing and engraftment of HSPCs was improved with intrabone transplantation compared to intravenous transfer 67, 68. ^89^Zr-oxine PET was used to further confirm the retention of HSPCs at the injection site in the iliac bone in rhesus macaques 69.

In another study, NK cells were expanded *ex vivo* with IL-2, per protocol used for human NK cell clinical trials, labeled with ^89^Zr-oxine, and tracked by ^89^Zr-oxine PET after autologous adoptive transfer, for preclinical evaluation of NK cell therapy against hematological malignancies such as leukemia. In this study, ^89^Zr-oxine PET revealed that transferred NK cells showed only minimal homing to the bone marrow, and most transferred NK cells distribute to the liver and spleen (Figure 2B) 64. This implies that for NK cells to be useful in treating bone marrow malignancies, NK cells must be engineered to track to the bone marrow in greater numbers than unmodified NK cells.

In humans, it has been known since the 1940s that glucocorticoid treatment rapidly decreases the number of eosinophils in peripheral blood within 1-2 hours of administration 70; however, it has been unclear where the eosinophils re-distribute in the body. To address this question, eosinophils purified from peripheral blood were labeled with ^89^Zr-oxine, transferred back to the animal, and imaging was performed before and after glucocorticoid treatment. ^89^Zr-oxine PET demonstrated a rapid increase of bone marrow accumulation of ^89^Zr-oxine labeled eosinophils over a four-hour period after glucocorticoid treatment 71. This demonstrates the high sensitivity of ^89^Zr-oxine PET to detect rapid changes in cell distribution in response to exogenous stimuli.

These results indicate that high-resolution cell tracking using ^89^Zr-oxine PET can also be successfully performed in non-human primates using clinical PET scanners, providing a rationale for the translation of cell tracking using ^89^Zr-oxine PET to human trials.

## Pitfalls and clinical translation of ^89^Zr-oxine PET

There are several pitfalls associated with ^89^Zr-oxine PET and other *ex vivo* labeling methods. One is the possible transfer of the label to phagocytes after the death of labeled cells. As observed in the use of iron nanoparticles for MRI 72, radioisotopes that are tightly bound to a cell structure or deposited in tissue could be transferred to tissue monocytes/macrophages by phagocytosis, resulting in non-specific signals. In this regard, studies using ^89^Zr-oxine so far have shown that ^89^Zr is released from dead cells and mostly cleared from the kidney. This process can be expedited by utilizing deferoxamine infusion as described below 55, 64. ^89^Zr-oxine does not passively transfer from labeled cells to neighboring cells 64.

Another pitfall is that there can be bone uptake from small amounts of released ^89^Zr after death of labeled cells 40, 55, 56. However, this can be again avoided by deferoxamine to chelate free ^89^Zr and enhance clearance from kidneys 55, 64. Deferoxamine is clinically used for iron overdose and thus potentially could be used safely during the imaging.

Before translation of ^89^Zr-oxine PET technology into the clinical setting, a number of important criteria must be met. There must be simplicity in the labeling method, low cytotoxicity in labeled cells, and no side effects to recipients of labeled cells. Cell labeling with ^89^Zr-oxine is straightforward and exerts minimal toxicity to labeled cells when optimal cell labeling doses were used. Although no clinical trials in humans have been performed, dosimetry data in non-human primates that received autologous NK cells showed very low radio-exposures to organs 64, and no clinical side effects were observed with autologous transfer of NK cells, CD34^+^ HPSCs 64 and eosinophils 71, confirming the safety of this method. Practically, labeling only a small fraction (*e.g.* 10 %) of the cells infused for cell-based therapy should be sufficient to obtain PET images with sufficiently high quality for cell tracking.

## Concluding remark

Here, we have reviewed our experience with cell labeling using ^89^Zr-oxine in preclinical models of *in vivo* tracking. Cell labeling with ^89^Zr-oxine can be performed in relatively simple steps. Once labeled, ^89^Zr is stably retained in cells until cell death. After cell death,^ 89^Zr is quickly liberated and generally cleared from the kidneys with enhanced clearance if deferoxamine is infused. ^89^Zr-oxine PET allows high-resolution tracking of transferred cells both in murine and non-human primate models for 1-2 weeks after transfer. These preclinical observations collectively provide a strong rationale for clinical translation of this technology.

## Abbreviations

CAR-T: chimeric antigen receptor T
HSPC: hematopoietic stem and progenitor cell
iPSC: induced pluripotent stem cell
MRI: magnetic resonance imaging
NK: natural killer
OVA: ovalbumin
PET: positron emission tomography
SPECT: single photon emission computed tomography
SPIO: superparamagnetic iron oxide
TCR: T cell receptor
TIL: tumor-infiltrating lymphocyte
^89^Zr: Zirconium-89
